# Lectures on Chemical Pathology

**Published:** 1902-12

**Authors:** 


**Lectures on Chemical Pathology.** By C. A. HERTER, M.D. Pp. xv., 461. London: Smith, Elder, & Co. 1902.

In publishing these lectures, which were delivered at the University and Bellevue Hospital Medical School, New York, the author hopes they will prove useful to students and practitioners of medicine who have not had the opportunity of keeping in touch with modern research in the field of chemical pathology. The opening chapter on the chemical defences of the organism against disease gives, on the whole, an excellent summary of present-day theories on these questions; we regret, however, that the work of Metschnikoff and the Metschnikoff school of investigators obtain so little notice in this section, being dismissed in a few lines.

The chapters on the chief food-stuffs and their fate in the body in health and disease; excessive fermentation in the digestive tract; the chemical pathology of gastric and intestinal digestion and of hepatic disease, are highly instructive; but the best section of all is, we think, that on diabetes.

While remembering that there are other excellent works dealing with these subjects, we cordially welcome this as an eminently scientific and most readable summary of recent investigations on chemical pathology.

